# Autistic adults have insight into their relative face recognition ability

**DOI:** 10.1038/s41598-024-67649-8

**Published:** 2024-08-01

**Authors:** Bayparvah Kaur Gehdu, Clare Press, Katie L. H. Gray, Richard Cook

**Affiliations:** 1 Department of Psychological Sciences, Birkbeck, University of London, London, UK; 2 Department of Experimental Psychology, University College London, London, UK; 3 Wellcome Centre for Human Neuroimaging, University College London, London, UK; 4 School of Psychology and Clinical Language Sciences, University of Reading, Reading, UK; 5 School of Psychology, University of Leeds, Leeds, LS2 9JT UK

**Keywords:** Face recognition, Twenty-item prosopagnosia index, Autism, Developmental prosopagnosia, Psychology, Human behaviour

## Abstract

The PI20 is a self-report questionnaire that assesses the presence of lifelong face recognition difficulties. The items on this scale ask respondents to assess their face recognition ability relative to the rest of the population, either explicitly or implicitly. Recent reports suggest that the PI20 scores of autistic participants exhibit little or no correlation with their performance on the Cambridge Face Memory Test—a key measure of face recognition ability. These reports are suggestive of a meta-cognitive deficit whereby autistic individuals are unable to infer whether their face recognition is impaired relative to the wider population. In the present study, however, we observed significant correlations between the PI20 scores of 77 autistic adults and their performance on two variants of the Cambridge Face Memory Test. These findings indicate that autistic individuals can infer whether their face recognition ability is impaired. Consistent with previous research, we observed a wide spread of face recognition abilities within our autistic sample. While some individuals approached ceiling levels of performance, others met the prevailing diagnostic criteria for developmental prosopagnosia. This variability showed little or no association with non-verbal intelligence, autism severity, or the presence of co-occurring alexithymia or ADHD.

## Introduction

Historically, lifelong face recognition difficulties were thought to be extremely rare^1^. Over the last twenty years, however, there has been growing appreciation that ‘congenital’ or ‘developmental’ prosopagnosia is far more common than was once believed^2–5^. Indeed, around 2% of the general population describe lifelong face recognition problems severe enough to disrupt their daily lives^6,7^. The incidence of lifelong face recognition difficulties is particularly high amongst autistic individuals, many of whom experience problems when asked to identify or match faces^8–11^.

Increasing awareness of these difficulties has fuelled the development of tools for the identification and assessment of face recognition impairments. One well-known measure is the Cambridge Face Memory Test (CFMT)^12,13^, a standardized objective test of face recognition ability that was developed to identify cases of developmental prosopagnosia. On each trial (72 in total), participants are asked to identify recently learned target faces from a line-up of three options (a target and two foils). The addition of view-point changes and high-spatial frequency visual noise increases task difficulty in the later stages. The CFMT has good internal reliability^12,13^ and correlates well with other measures of face identification^14^.

A second measure developed to aid the identification of developmental prosopagnosia is the Twenty Item Prosopagnosia Index (PI20)^15–17^. This self-report questionnaire was designed to provide standardized self-report evidence of face recognition difficulties, to complement diagnostic evidence obtained from objective computer-based assessments such as the CFMT. The PI20 comprises 20 statements describing face recognition experiences drawn from qualitative and quantitative descriptions of individuals with lifelong face recognition difficulties. Respondents (typically adults) rate how well each statement describes their own experiences on a 5-point scale. Scores can range from 20 to 100. A score of 65 or higher is thought to indicate the likely presence of face recognition impairment. The PI20, originally written in English, has been translated into multiple languages (e.g., Italian, Portuguese, Danish, Japanese & Mandarin) and applied in various cultural contexts^18–22^. The twenty items comprising the PI20 can be viewed in the supplementary materials (Table S1).

The items on the PI20 ask respondents to assess their face recognition ability relative to the rest of the population, either explicitly (e.g., My face recognition ability is worse than most people; I am better than most people at putting a ‘name to a face’; I have to try harder than other people to memorize faces) or implicitly (e.g., When people change their hairstyle or wear hats, I have problems recognizing them; when I was at school, I struggled to recognize my classmates). There has been considerable debate about whether participants have the necessary insight into their relative face recognition ability to provide meaningful responses to such items ^23–26^. However, there is now strong evidence that the PI20 scores of non-autistic participants correlate with their performance on objective measures of face recognition accuracy^15–17,27^. While participants may lack fine-grained insight into their face recognition ability (e.g., whether they fall within the 45th or 55th percentile), these findings suggest that respondents have enough insight to provide meaningful responses on the PI20; i.e., they appear to have some idea whether their face recognition is impaired or unimpaired.

This may not be true of autistic individuals, however. Minio-Paluello and colleagues^28^ reported that the PI20 scores of autistic adults (*N* = 63) exhibited little or no correlation with their performance on the CFMT—a key objective test of face recognition ability. A similar result was described by Stantić and colleagues^10^. In this study, the authors observed a non-significant correlation of *r* = − 0.17 between the PI20 scores of 31 autistic adults and their performance on the CFMT. If found to be robust, these results have important theoretical implications: they raise the possibility that face recognition in autism may be subject to a metacognitive deficit, whereby autistic individuals are unable to infer whether (or not) their face recognition ability is impaired relative to the wider population. There is also an important substantive implication. These results suggests that the PI20 may not be suitable for screening autistic participants for face recognition difficulties. This would be a non-trivial limitation, not least because face recognition difficulties appear to be far more prevalent in the autistic population than in the non-autistic population^8–11^.

There are several reasons to be cautious when interpreting these findings, however. First, previous research suggests that metacognitive differences in autistic adults tend to be small and subtle, if observed at all^29^. Second, the study described by Stantić et al.^10^ was not designed to examine individual differences. Any conclusions about face recognition variability and correlations therewith, are limited by the relatively small size of the study’s autistic sample (*N* = 31). Correlation estimates obtained with small samples are notoriously unstable^30^. Third, both results were obtained using the original version of the CFMT (the CFMT-O)^13^. This version of the CFMT is now easily accessible online; it is hosted by several websites, and various prosopagnosia forums and pop-science resources link to this test. Consequently, many individuals with face recognition difficulties have attempted the CFMT-O on multiple occasions^31^. Where practice benefits arise, participants may achieve higher scores than might be expected based on their PI20 score.

In light of the foregoing observations, we were keen to re-examine the relationship between the PI20 scores and CFMT performance of autistic individuals. To this end, a group of 77 autistic participants completed the PI20 questionnaire and two variants of the CFMT: the original (CFMT-O)^13^ and the Australian (CFMT-A)^12^ versions. The CFMT-O and CFMT-A share an identical format and differ only in terms of the (White male) facial identities used. Unlike the CFMT-O, however, the CFMT-A is not widely available to the members of the general public.

It has been noted previously that the face recognition abilities of autistic participants vary widely^8–11,28^. At present, however, little is known about the nature and origin of this variability. Some of this variance might be explained by differences in autism severity^32^. However, performance on face processing tasks may also be affected by differences in non-verbal intelligence^33^ and the presence of co-occurring conditions, notably alexithymia^34,35^ and attention-deficit-hyperactivity disorder (ADHD)^36,37^. We therefore took this opportunity to explore which of these factors—if any—predicted face recognition performance in our autistic sample.

## Methods

### Participants

Seventy-seven participants with a clinical diagnosis of autism (*M*_age_ = 35.99 years; *SD*_*a*ge_ = 11.60 years) were recruited via www.ukautismresearch.org. All autistic participants had received an autism diagnosis (e.g., Autism Spectrum Disorder, Asperger’s Syndrome) from a clinical professional (General Practitioner, Neurologist, Psychiatrist or Clinical Psychologist) based in the U.K. All participants in the autistic group also reached cut-off (a score of 32) on the Autism Spectrum Quotient (AQ)^38^. The mean AQ score of the participants was 42.45 (*SD* = 4.17). To be eligible, participants also had to be aged between 18 and 60, speak English as a first language and have normal or corrected-to-normal visual acuity. All participants were required to be a current U.K. resident.

Of the 16 individuals who described their sex as male, 13 described their gender identity as male, 2 identified as non-binary and 1 identified as female. Of the 61 individuals who described their sex as female, 48 described their gender identity as female, 9 identified as non-binary, 1 as male and 3 preferred not to say. Seventy-six of the 77 participants identified as White (73: White-British, 1: White Irish, 2: White-Other). One participant did not specify their ethnicity. Sixty-eight of the participants were right-handed, while 9 were left-handed.

Data collection for the study took place between June and August 2023. At the outset, our aim was i) to recruit as many participants as possible during this period, and ii) to stop data collection at the end of August provided a minimum sample size of *N* = 62 had been reached. A sample of *N* = 62 yields a 90% chance of detecting a correlation of *r* = 0.40 between PI20 scores and CFMT performance. Our final sample (*N* = 77) comfortably exceeded this minimum.

Ethical clearance was granted by the Departmental Ethics Committee for Psychological Sciences, Birkbeck, University of London and the experiment was conducted in line with the ethical guidelines laid down in the 6th (2008) Declaration of Helsinki. All participants gave informed consent before taking part.

### Measures

The principal aim of the study was to elucidate the relationship between participants’ PI20 scores and their performance on the CFMT. To this end, all participants completed the PI20 questionnaire^16^ and two versions of the CFMT: the CFMT-O^13^ and the CFMT-A^12^. All participants completed the PI20 before attempting the CFMTs. Participants also completed the AQ to confirm their eligibility for the study. In addition to these measures, all participants completed a self-report measure of alexithymia severity: the Twenty-item Toronto Alexithymia Scale (TAS20)^39,40^, a self-report measure of ADHD traits: the Adult ADHD Self-Report Scale (ASRS)^41^, and a matrix reasoning task (MRT) to assess their non-verbal intelligence.

The TAS20 comprises 20 statements that relate to one’s ability to describe and identify emotions and interoceptive sensations. Respondents indicate to what extent each statement applies to them on a 5-point scale. Scores can range from 20 to 100, with higher scores indicative of more alexithymic traits. A score of 61 or higher is thought to index clinically significant levels of alexithymia. The TAS20 has good psychometric properties^39^ and is widely used to assess the presence of alexithymia in autistic and non-autistic individuals^34^.

The ASRS is a self-report questionnaire that assesses the presence of traits associated with inattention, hyperactivity, and impulsivity. The ASRS consists of two parts: Part A is a 6-item screener that has been shown to effectively discriminate clinical cases of adult ADHD from non-cases^42^. Each response is scored as either 0 or 1, thus screener scores can range from 0 to 6. A score of 4 or above is thought to be associated with clinically significant levels of ADHD traits. Part B consists of 12 follow-up items that can be used to probe symptomology. Part B was not employed here.

The MRT employed consists of forty items selected from The Matrix Reasoning Item Bank^43^. Participants were given 30 s to complete each puzzle by selecting the correct answer from 4 options. Participants responded using keyboard number keys (1–4), were given a 5-s warning before the end of each trial, and received no feedback. Each participant attempted all forty items. Participants had to complete 3 practice trials correctly before beginning the test. We have employed this measure in previous studies of social perception in autism^8,35^. Based on a sample of 100 non-autistic adults (*M*_age_ = 34.90; *SD*_*a*ge_ = 10.16), we estimate the test–retest reliability of this measure to be *r*_p_ = 0.727 (see Supplementary Material). All data reported here were collected online. Both versions of the CFMT and the matrix reasoning test were administered via Gorilla Experiment Builder^44^.

### Statistical procedures

The correlational analyses described below (all α = 0.05, two-tailed) were conducted using Pearson’s *r* (*r*_p_) and Spearman’s rho (*r*_s_)*.* The comparison of autistic subgroups was assessed using independent samples *t*-tests (α = 0.05, two-tailed). For each *t*-test we also provide the associated Bayes factor (BF), calculated in JASP^45^ with default prior width. We interpret BFs of less than 3.0 as anecdotal evidence for the null hypothesis. BFs of greater than 3.0 are treated as substantial evidence for the null hypothesis^46^. The data supporting all the analyses described are available via the Open Science Framework (https://osf.io/tesk5/).

## Results

The mean scores obtained for each measure are shown in Table 1. As expected, we saw strong correlation between performance on the CFMT-O and CFMT-A [*N* = 77, *r*_p_ = 0.744, *p* < 0.001], underscoring the good psychometric properties of our two dependent measures. We also observed a number of significant correlations between our predictor variables (Table 1). Reassuringly, several of these relationships are predicted by the existing literature^34,47,48^, including the AQ-TAS20 correlation [*N* = 77, *r*_p_ = 0.526, *p* < 0.001] and the AQ-ASRS correlation [*N* = 77, *r*_p_ = 0.322, *p* = 0.004]. The mean PI20 score (*M* = 62.43) accords well with the mean PI20 score described by Stantić and colleagues^10^ (*M* = 63.30) but is a little higher than that reported by Minio-Paluello and colleagues^28^ (*M* = 55.5).

**Table 1:** Mean performance of the autistic sample on the measures employed in the study and their respective correlations (*r*_p_).

Variable	*M* (*SD*)	α	1	2	3	4	5	6	7
1. CFMT-O	67.95 (15.80)	0.912	–						
2. CFMT-A	70.67 (15.22)	0.909	0.744***	–					
3. PI20	62.43 (20.19)	0.947	− 0.486***	− 0.464***	–				
4. AQ	42.45 (4.17)	0.699	− 0.190	− 0.173	0.301**	–			
5. TAS20	67.82 (12.23)	0.865	− 0.177	− 0.252*	0.292**	0.526***	–		
6. MRT	25.32 (5.95)	0.817	− 0.090	0.001	0.214	− 0.089	0.046	–	
7. ASRS	4.21 (1.67)	0.680	0.077	0.064	0.267*	0.322**	0.453***	0.090	–

CFMT-O: Cambridge Face Memory Test – Original version. CFMT-A: Cambridge Face Memory Test – Australian version. PI20: Twenty-item Prosopagnosia Index. AQ: Autism-Spectrum Quotient. TAS20: Toronto Alexithymia Scale. MRT: Matrix Reasoning Test. ASRS: Adult ADHD Self-Report Scale. *p < 0.05; **p < 0.01, ***p < 0.001.

### Does the autistic sample exhibit poor face recognition?

The present study had two principal aims: first, to establish whether or not the PI20 scores of autistic adults correlate with their CFMT performance. Second, to explore whether differences in non-verbal intelligence and the presence of co-occurring conditions (alexithymia and ADHD) account for the enormous variability in face recognition ability seen in the autistic population. Thus, the focus of our investigation is the variability in face recognition performance observed within the autistic sample.

**Figure 1:**
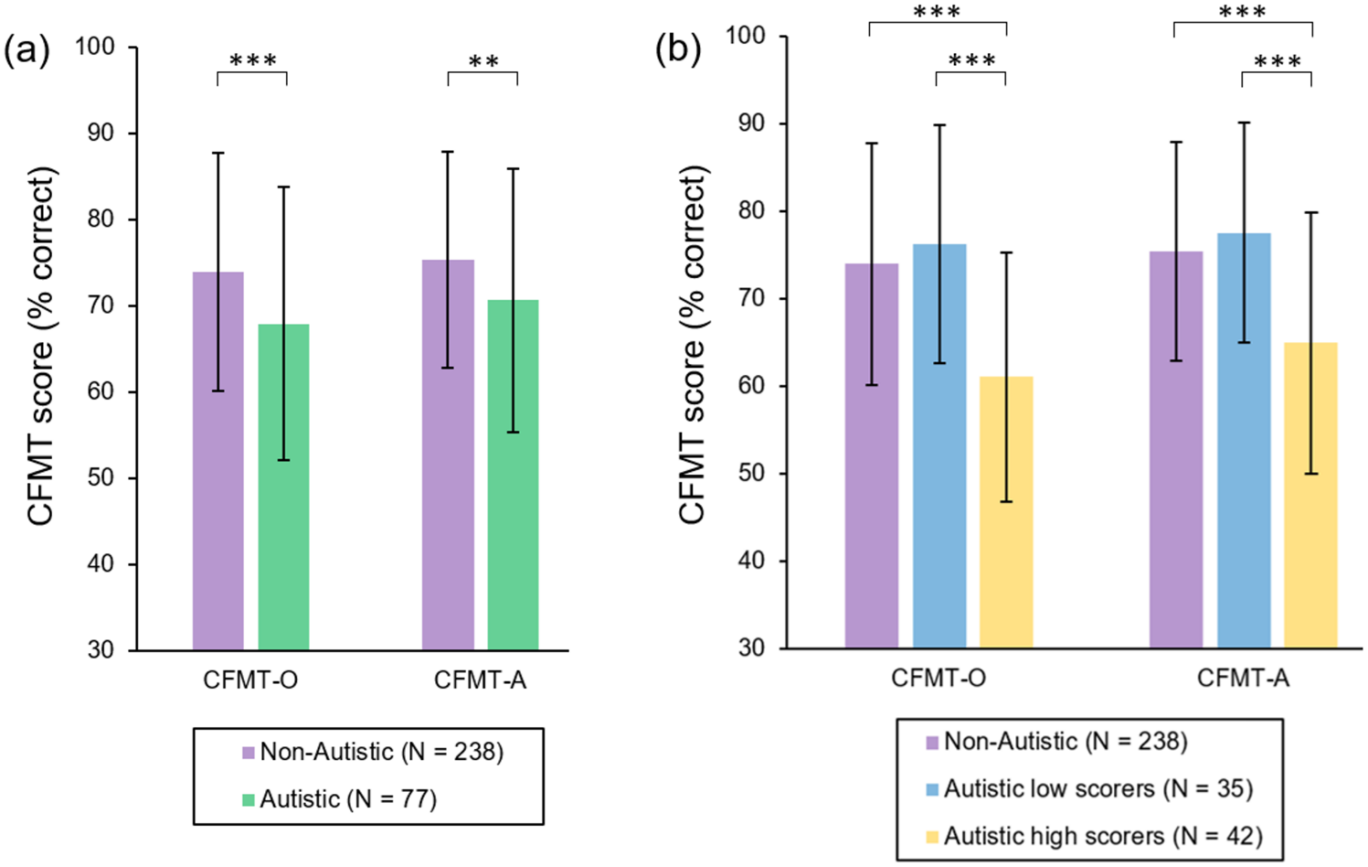
(**a**) Mean scores on the CFMT-O and CFMT-A for the autistic sample. (**b**) Mean scores on the CFMT-O and CFMT-A for those autistic participants who reached the PI20 cut-off score (high-scorers) and those who did not (low-scorers). The non-autistic comparison data illustrated in both panels is taken from Tsantani et al.^17^. **p ≤ 0.01, ***p ≤ 0.001. Error bars denote ± 1SD.

At the outset of our analyses, however, we first sought to evaluate the overall performance of the autistic sample on the CFMT-O (*M* = 67.95, *SD* = 15.80) and CFMT-A (*M* = 70.67, *SD* = 15.22). For this purpose, we employed comparison data reported by Tsantani et al.^17^ (Fig. 1a). These data were obtained from 238 non-autistic individuals (131 females, 104 males, 3 non-binary; *M*_age_ = 36.56, *SD*_age_ = 11.72), who completed online versions of the CFMT-O (*M* = 73.96, *SD* = 13.77) and CFMT-A (*M* = 75.37, *SD* = 12.48) under similar conditions. The participants in this sample were recruited via Prolific (www.prolific.com). Thirteen of the 238 participants (5.46%) reached the PI20 cut-off score of 65 (*M* = 44.85, *SD* = 10.70).

As expected, the scores of the autistic participants in our sample were significantly below those seen in this comparison sample, both for the CFMT-O [*t*(313) = 3.207, *d* = 0.420, *p* = 0.001, BF_01_ = 0.057] and the CFMT-A [*t*(110.97) = 2.453, *p* = 0.016, *d* = 0.356, BF_01_ = 0.221]. Note, for this latter comparison it was necessary to correct the degrees of freedom because the variance in our autistic sample was greater than that seen in the non-autistic comparison data [*F*(1, 313) = 5.387, *p* = 0.021]. The fact that the CFMT scores of our autistic sample tended to be lower than those of the non-autistic comparison sample accords well the existing literature^8–11^. This finding suggests that, in terms of face recognition ability, our autistic sample is broadly comparable with autistic samples described elsewhere.

### Do PI20 scores predict CFMT scores?

**Figure 2:**
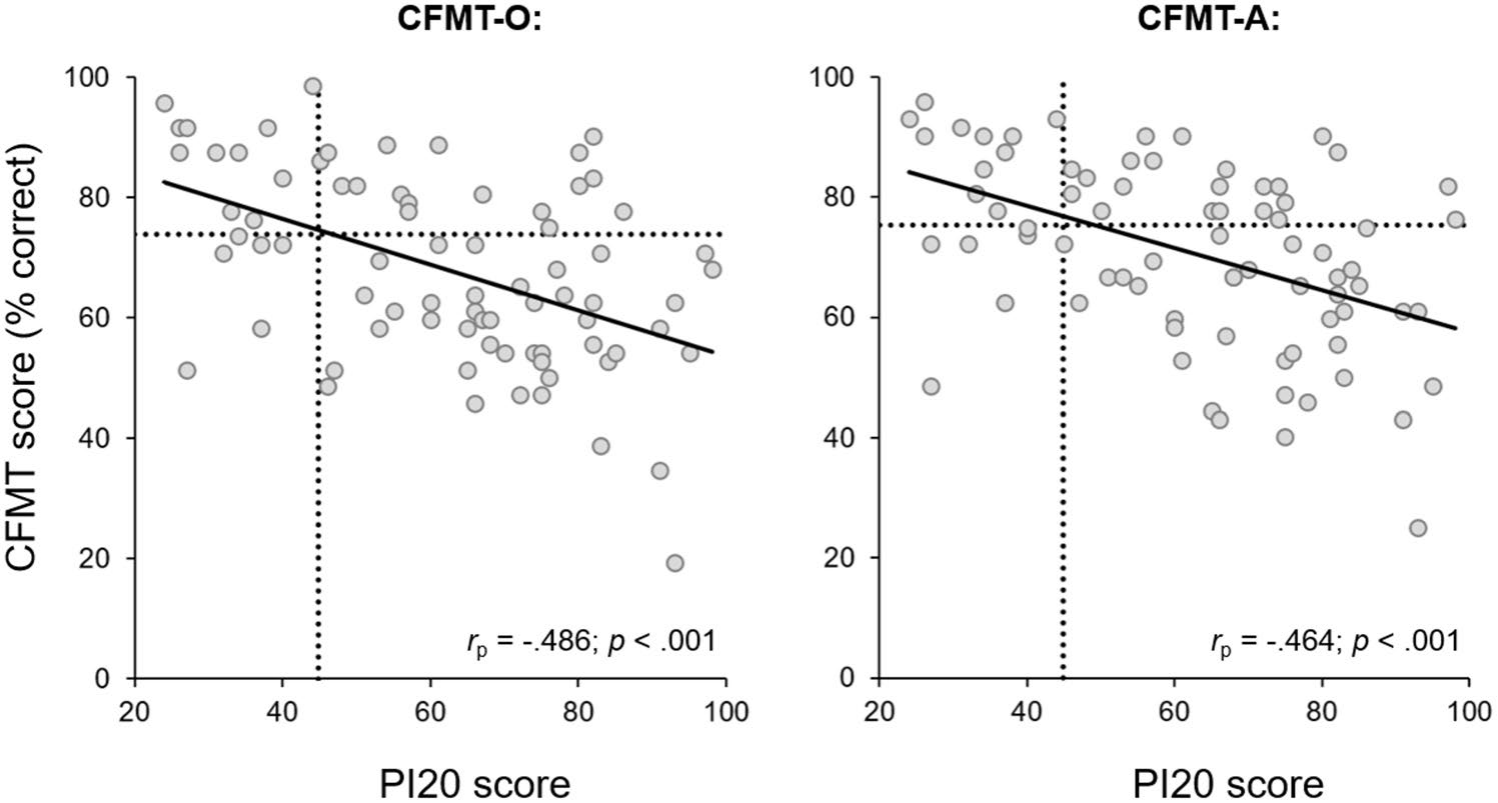
Scatterplots of the relationship between PI20 scores and performance on the CFMT-O (left) and the CFMT-A (right). The solid lines depict the linear trends. The dashed lines depict the mean performance of the non-autistic sample described by Tsantani et al.^17^.

Next, we sought to determine whether the PI20 scores of our autistic participants were predictive of their CFMT performance. To begin, we examined the simple correlations between participants’ PI20 and CFMT scores. Contrary to the findings of Minio-Paluello et al.^28^ and Stantić et al.^10^, we observed significant correlation between PI20 scores and performance on both the CFMT-O [*N* = 77, *r*_p_ = − 0.486, *p* < 0.001] and CFMT-A [*N* = 77, *r*_p_ = − 0.464, *p* < 0.001] (Fig. 2). Similar correlations were seen when the raw scores were transformed into ranks for both the CFMT-O [*N* = 77, *r*_s_ = − 0.435, *p* < 0.001] and CFMT-A [*N* = 77, *r*_s_ = − 0.469, *p* < 0.001].

We also conducted a complementary subgroup analysis based on the established PI20 cut-off of 65. We split our sample of 77 autistic participants into those who met the cut-off (*N* = 42, *M*_age_ = 37.40, *SD*_age_ = 11.61) and those who did not (*N* = 35, *M*_age_ = 34.29, *SD*_age_ = 11.51). The autistic participants who met the PI20 cut-off achieved significantly lower scores than those who did not on both the CFMT-O [low-scorers: *M* = 76.23, *SD* = 13.59; high-scorers: *M* = 61.04, *SD* = 14.21; *t*(75) = 4.762, *p* < 0.001, *d* = 1.090, BF_01_ < 0.001] and the CFMT-A [low-scorers: *M* = 77.54, *SD* = 12.60; high-scorers: *M* = 64.95, *SD* = 14.97; *t*(75) = 3.946, *p* < 0.001, *d* = 0.903, BF_01_ = 0.007] (Fig. 1b). Moreover, the autistic participants who met the PI20 cut-off performed worse on the CFMT-O [*t*(278) = 5.576, *p* < 0.001, *d* = 0.933, BF_01_ < 0.001] and CFMT-A [*t*(278) = 4.835, *p* < 0.001, *d* = 0.809, BF_01_ < 0.001] than the non-autistic participants tested by Tsantani and colleagues^17^ (Fig. 1b). In contrast, the CFMT-O scores [*t*(271) = − 0.914, *p* = 0.362, *d* = − 0.165, BF_01_ = 3.556] and CFMT-A scores [*t*(271) = − 0.960, *p* = 0.338, *d* = − 0.174, BF_01_ = 3.419] of the autistic participants who did not meet the PI20 cut-off did not differ significantly from the comparison distributions described by Tsantani et al.^17^.

### Do co-occurring alexithymia and ADHD predict face recognition in autism?

**Figure 3:**
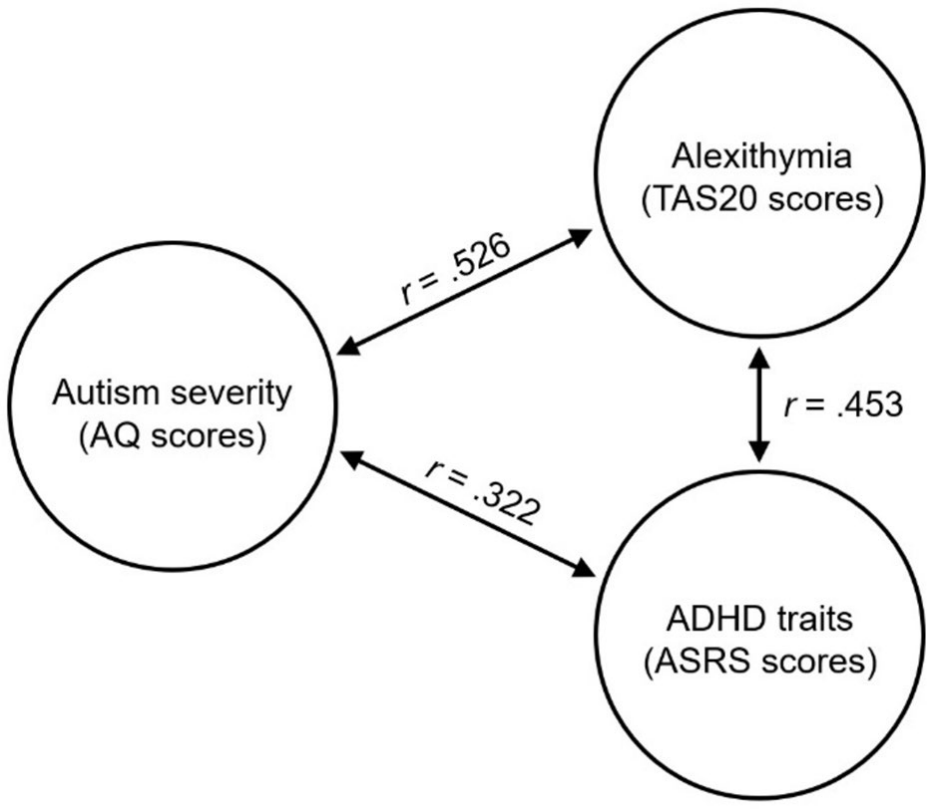
Simple correlations observed between autism severity (inferred from scores on the AQ questionnaire), levels of alexithymia (inferred from TAS20 scores), and the presence of ADHD traits (inferred from the ASRS screener). All correlations are significant at *p* < 0.001 (*N* = 77).

There was some correlation between scores on the TAS20—a measure of alexithymia—and performance on the CFMT-A [*N* = 77, *r*_p_ = − 0.252, *p* = 0.027]. We also observed a significant correlation between TAS20 scores and average performance on the CFMT-O and CFMT-A [*N* = 77, *r*_p_ = − 0.229, *p* = 0.045]. However, we failed to observe a significant relationship with CFMT-O scores independently [*N* = 77, *r*_p_ = − 0.177, *p* = 0.125]. We also note that the significant TAS20-CFMT correlations described above do not survive correction for multiple comparisons. No significant correlation was observed between scores on the ASRS—a measure of ADHD traits—and either CFMT-O scores [*N* = 77, *r*_p_ = 0.077, *p* = 0.504] or CFMT-A scores [*N* = 77, *r*_p_ = 0.064, *p* = 0.580]. Interestingly, we observed a noteworthy correlation between TAS20 and ASRS scores [*N* = 77, *r*_p_ = 0.453, *p* < 0.001]; i.e., those autistic participants who reported high levels of alexithymic traits also reported higher levels of ADHD traits (Fig. 3).

Like the PI20, both the TAS20 and the ASRS have established cut-offs, associated with clinically significant levels of alexithymia and ADHD traits, respectively. We therefore examined whether subgroup analyses of TAS20 and ASRS scores would reveal evidence of a predictive relationship with CFMT. Of the 77 autistic participants, 59 met the TAS20 cut-off for clinically significant levels of alexithymia, while 18 did not. Those who met cut-off and those who did not, did not differ in their scores on the CFMT-O [low-scorers: *M* = 70.22, *SD* = 14.78; high-scorers: *M* = 67.26, *SD* = 16.15; *t*(75) = 0.694, *p* = 0.490, *d* = 0.187, BF_01_ = 3.016] or on the CFMT-A [low-scorers: *M* = 74.15, *SD* = 11.32; high-scorers: *M* = 69.61, *SD* = 16.60; *t*(75) = 1.110, *p* = 0.271, *d* = 0.299, BF_01_ = 2.212]. Similarly, 51 autistic participants met the ASRS cut-off for clinically significant ADHD traits, while 26 did not. Once again, there was little sign that CFMT-O scores [low-scorers: *M* = 66.61, *SD* = 18.64; high-scorers: *M* = 68.63, *SD* = 14.29; *t*(75) = 0.527, *p* = 0.600, *d* = 0.127, BF_01_ = 3.586] or CFMT-A scores [low-scorers: *M* = 71.53, *SD* = 16.29; high-scorers: *M* = 70.23, *SD* = 14.80; *t*(75) = 0.351, *p* = 0.727, *d* = 0.084, BF_01_ = 3.831] differed across these subgroups.

### Is face recognition in autism affected by non-verbal intelligence or autism severity?

No significant correlation was observed between AQ scores and CFMT-O scores [*N* = 77, *r*_p_ = − 0.190, *p* = 0.098] or between AQ scores and CFMT-A scores [*N* = 77, *r*_p_ = − 0.173, *p* = 0.131]. Note, however, meeting the AQ cut-off score was part of the study inclusion criteria; hence, all 77 autistic participants had an AQ score of 33 or higher. Similarly, no significant correlation was observed between MRT scores and CFMT-O scores [*N* = 77, *r*_p_ = − 0.090, *p* = 0.436] or between MRT scores and CFMT-A scores [*N* = 77, *r*_p_ = 0.001, *p* = 0.992]. In sum, we find no evidence in our data that non-verbal intelligence or autism severity influence the face recognition abilities of autistic participants.

## General discussion

There is now considerable evidence that the PI20 scores of non-autistic participants correlate with their performance on objective measures of face recognition accuracy^15–17,27^. These findings suggest that respondents have enough insight into their relative face recognition ability to provide meaningful responses on the PI20. Recently, however, Minio-Paluello et al.^28^ reported that the PI20 scores of autistic participants (*N* = 66) exhibited little or no correlation with their performance on the CFMT. A similar finding was described by Stantić et al.^10^, albeit with a smaller sample (*N* = 31). These reports are potentially important because they suggest the possibility that autistic individuals may experience a metacognitive deficit, whereby they are unable to infer whether (or not) their face recognition ability is impaired. Moreover, these results raise the possibility that the PI20 may be unsuitable for screening autistic participants for face recognition difficulties.

Contrary to these reports, however, we find clear evidence of association between the PI20 scores of autistic participants (*N* = 77) and their performance on the CFMT-O and the CFMT-A. This association was evident both in simple correlation analyses, and in subgroup analyses where the autistic sample was split into those who met the established cut-off for developmental prosopagnosia, and those who did not. The mean score of those autistic participants who met cut-off was ~ 15% and ~ 12.5% lower than those that did not, on the CFMT-O and CFMT-A, respectively. Indeed, those autistic participants who did not meet the PI20 cut-off exhibited similar levels of performance to a non-autistic comparison sample described previously^17^. Together, these analyses provide clear evidence that the PI20 scores of autistic participants predict their CFMT performance.

The most likely explanation for the failure of Stantić et al.^10^ to detect a correlation between scores on the PI20 and the CFMT is the relatively small size of their autistic group (*N* = 31). As we allude to in the introduction, (1) this study was not designed to examine the individual differences seen within the autistic population, and (2) correlation estimates obtained with small samples are notoriously unstable^30^. Post-hoc power analysis indicates there is a 38% chance of failing to detect a significant correlation of *r* = 0.40 with a sample of this size (α = 0.05, two-tailed).

Assuming the authors scored the PI20 correctly, the null correlation described by Minio-Paluello et al.^28^ is harder to explain. One relevant factor may be the wide range of general cognitive abilities present in their autistic sample (*N* = 63). As a self-report scale, the PI20 has relatively high verbal demands potentially making it unsuitable for individuals with intellectual disability. Moreover, five of the twenty items are reverse scored. Respondents must therefore read individual items carefully to respond appropriately. If some of the participants tested by Minio-Paluello et al.^28^ struggled to interpret scale items, and were unable to respond appropriately, this might also explain why the mean PI20 score was lower than that reported here and elsewhere^10^.

It is now beyond doubt that the face recognition abilities of autistic participants vary enormously^8–11,28^. Once again, we saw evidence of this variability in our sample. On the one hand, 13 of our 77 autistic participants (16.9%) scored 65 or higher on the PI20 and scored less than 60% on both versions of the CFMT. These individuals would meet the diagnostic criteria for developmental prosopagnosia employed by the vast majority of research groups^13,49^. On the other hand, 10 of the 77 autistic participants (13.0%) scored 85% or higher on both tests, suggestive of excellent face recognition^12,17^.

There was little sign in our data that variability in face recognition ability is attributable to differences in non-verbal intelligence (as measured by MRT score), autism severity (as measured by AQ score), or the presence co-occurring ADHD traits (as measured by ASRS score). There was some hint of a potential relationship between the presence of co-occurring alexithymia and face recognition ability: TAS20 scores were negatively correlated with performance on the CFMT-A and with average CFMT performance. However, TAS20 scores did not exhibit significant correlation with CFMT-O scores independently, and the foregoing correlations do not survive correction for multiple-comparisons.

What should we make of this variability? We favour the view that, like alexithymia and ADHD, developmental prosopagnosia is a neurodevelopmental condition that can occur independently of autism, but that also frequently co-occurs with autism^4,8,51,52^. This view not only accounts for the severe lifelong face recognition problems seen in some autistic individuals, but also explains why many other autistic individuals exhibit excellent face recognition. Moreover, this account accords with the prevailing view that the co-occurrence of neurodevelopmental conditions is the ‘norm’ rather than the exception^34,47,48,53–55^. Given what we know about neurodevelopmental conditions more broadly, it would be hugely surprising if the incidence of developmental prosopagnosia was not elevated in the autistic population.

Recently, some authors have rejected this account citing evidence that autistic samples still exhibit below-average face recognition when those who meet the diagnostic criteria for prosopagnosia are removed^11^. However, this critique overlooks two issues. First, diagnostic assessments for developmental prosopagnosia are imperfect^26^. Many autistic individuals with severe co-occurring prosopagnosia may fail to meet diagnostic thresholds simply because of measurement error. Second, the severity of developmental prosopagnosia is thought to vary^56^. While some autistic individuals may experience severe developmental prosopagnosia, others may experience relatively mild forms. These latter individuals may fail to meet conservative diagnostic criteria for developmental prosopagnosia, but still exhibit below average face recognition.

While it was not the focus of our study, we observed a striking correlation between the presence of alexithymia and ADHD traits in our autistic participants. The fact that those autistic participants who report high levels of alexithymia also tend to report high levels of ADHD traits is potentially significant for understanding socio-cognitive differences in autism. In recent years, there has been increasing suggestion that many social perception difficulties traditionally attributed to autism—such as atypical interpretation of facial expressions^35,57^ and reduced eye-region fixations^50,58^—may actually be products of co-occurring alexithymia. Likewise, there is some suggestion that other socio-cognitive differences attributed to autism—for example, atypical attentional cueing by gaze direction^37^—may be partly attributable to co-occurring ADHD. To date, however, authors have tended to assess the presence of *either* co-occurring alexithymia *or* co-occurring ADHD. In future, it may prove valuable to establish the extent to which these constructs exert independent or interactive effects in these domains.

### Limitations and future directions

We note that 61 of our 77 participants described their sex as female. Conversely, the majority of the autistic population are thought to identify as male^59^. This is not the first time that a high proportion of female participants has been seen where studies have sought to recruit autistic participants online^60^. Unlike participant age^61^, the sex/gender of observers is not thought to exert a strong influence on face recognition ability^62^. However, we acknowledge the need to replicate the present findings in a sample more representative of the wider autistic community.

It is important that future research ascertain if/how other measures of meta-cognitive performance—such as estimates of meta *c* and meta *d* inferred from type-II signal detection tasks^63,64^—relate to participants’ responses on the PI20. For example, one might hypothesize that the PI20 scores of those with a higher meta c ought to correspond more closely to objective face recognition performance. It might also be interesting to examine how autistic and non-autistic individuals acquire insight into their relative face recognition abilities (e.g., What kinds of face recognition errors are salient? What have individuals been told about face recognition in autism?).

## Conclusion

Contrary to recent reports, we observed significant correlation between PI20 scores and performance on both the CFMT-O and CFMT-A in autistic adults. This finding indicates that autistic individuals are able to infer whether (or not) their face recognition ability is impaired and confirms that the PI20 can be used to screen autistic participants for face recognition difficulties. Consistent with previous research, the face recognition performance within our autistic sample varied considerably. While some individuals approached ceiling levels of recognition accuracy, others met the prevailing diagnostic criteria for developmental prosopagnosia. This variability showed little or no association with non-verbal intelligence, autism severity, or the presence of co-occurring alexithymia or ADHD.

### Supplementary Information

Supplementary Information.

## Data Availability

The data supporting all the analyses is available here: https://osf.io/tesk5/.

## References

[1] McConachie, H. R. Developmental prosopagnosia. A single case report. *Cortex* **12**, 76–82 (1976). 1261287 10.1016/S0010-9452(76)80033-0

[2] Wilmer, J. B. Individual differences in face recognition: A decade of discovery. *Curr. Dir. Psychol. Sci.* **26**, 225–230 (2017). 10.1177/0963721417710693

[3] Behrmann, M. & Avidan, G. Congenital prosopagnosia: Face-blind from birth. *Trends Cogn. Sci.* **9**, 180–187 (2005). 15808500 10.1016/j.tics.2005.02.011

[4] Cook, R. & Biotti, F. Developmental prosopagnosia. *Curr. Biol.* **26**, R312–R313 (2016). 27115682 10.1016/j.cub.2016.01.008

[5] Duchaine, B. & Nakayama, K. Developmental prosopagnosia: A window to content-specific face processing. *Curr. Opin. Neurobiol.* **16**, 166–173 (2006). 16563738 10.1016/j.conb.2006.03.003

[6] Kennerknecht, I. *et al.* First report of prevalence of non-syndromic hereditary prosopagnosia (HPA). *Am. J. Med. Genet.* **140A**, 1617–1622 (2006). 10.1002/ajmg.a.31343 16817175

[7] Kennerknecht, I., Ho, N. Y. & Wong, V. C. N. Prevalence of heriditary prosopagonsia (HPA) in Hong Kong Chinese population. *Am. J. Med. Genet.* **146A**, 2863–2870 (2008). 18925678 10.1002/ajmg.a.32552

[8] Gehdu, B. K., Gray, K. L. & Cook, R. Impaired grouping of ambient facial images in autism. *Sci. Rep.* **12**, e6665 (2022). 10.1038/s41598-022-10630-0 PMC9035147 35461345

[9] Hedley, D., Brewer, N. & Young, R. Face recognition performance of individuals with Asperger syndrome on the Cambridge Face Memory Test. *Autism Res.* **4**, 449–455 (2011). 22162360 10.1002/aur.214

[10] Stantić, M., Ichijo, E., Catmur, C. & Bird, G. Face memory and face perception in autism. *Autism*, 13623613211027685 (2021). 10.1177/13623613211027685 PMC8750147 34160282

[11] Kamensek, T., Susilo, T., Iarocci, G. & Oruc, I. Are people with autism prosopagnosic? *Autism Res*. (2023). 10.1002/aur.3030 37740564

[12] McKone, E. *et al.* Face ethnicity and measurement reliability affect face recognition performance in developmental prosopagnosia: Evidence from the Cambridge face memory test-Australian. *Cogn. Neuropsychol.* **28**, 109–146 (2011). 22122116 10.1080/02643294.2011.616880

[13] Duchaine, B. & Nakayama, K. The Cambridge Face Memory Test: results for neurologically intact individuals and an investigation of its validity using inverted face stimuli and prosopagnosic participants. *Neuropsychologia* **44**, 576–585 (2006). 16169565 10.1016/j.neuropsychologia.2005.07.001

[14] Biotti, F., Gray, K. L. H. & Cook, R. Is developmental prosopagnosia best characterised as an apperceptive or mnemonic condition?. *Neuropsychologia* **124**, 285–298 (2019). 30502377 10.1016/j.neuropsychologia.2018.11.014

[15] Gray, K. L. H., Bird, G. & Cook, R. Robust associations between the 20-item prosopagnosia index and the Cambridge Face Memory Test in the general population. *Royal Society Open Sci.* **4**, 160923 (2017). 10.1098/rsos.160923 PMC5383837 28405380

[16] Shah, P., Gaule, A., Sowden, S., Bird, G. & Cook, R. The 20-item prosopagnosia index (PI20): A self-report instrument for identifying developmental prosopagnosia. *Royal Society Open Sci.* **2**, 140343 (2015). 10.1098/rsos.140343 PMC4632531 26543567

[17] Tsantani, M., Vestner, T. & Cook, R. The Twenty Item Prosopagnosia Index (PI20) provides meaningful evidence of face recognition impairment. *Royal Society Open Sci.* **8**, e202062 (2021). 10.1098/rsos.202062 PMC8564608 34737872

[18] Estudillo, A. J. & Wong, H. K. Associations between self-reported and objective face recognition abilities are only evident in above-and below-average recognisers. *PeerJ* **9**, e10629 (2021). 33510971 10.7717/peerj.10629 PMC7808263

[19] Tagliente, S. *et al.* Self-reported face recognition abilities moderately predict face-learning skills: Evidence from Italian samples. *Heliyon* **9**, e14125 (2023). 36915548 10.1016/j.heliyon.2023.e14125 PMC10006496

[20] Nørkær, E. *et al.* The Danish version of the 20-Item prosopagnosia index (PI20): Translation, validation and a link to face perception. *Brain Sciences* **13**, e337 (2023). 10.3390/brainsci13020337 PMC9954571 36831880

[21] Oishi, Y., Aruga, K. & Kurita, K. (2024), Relationship between face recognition ability and anxiety tendencies in healthy young individuals: A prosopagnosia index and state-trait anxiety inventory study. *Acta Psychol.* **245**, e104237 (2024). 10.1016/j.actpsy.2024.104237 38537601

[22] Ventura, P., Livingston, L. A. & Shah, P. Adults have moderate-to-good insight into their face recognition ability: Further validation of the 20-item Prosopagnosia Index in a Portuguese sample. *Quart. J. Exp. Psychol.* **71**, 2677–2679 (2018). 10.1177/1747021818765652 PMC6293437 29504464

[23] Bobak, A. K., Mileva, V. R. & Hancock, P. J. Facing the facts: Naive participants have only moderate insight into their face recognition and face perception abilities. *Quart. J. Exp. Psychol.* **72**, 872–881 (2019). 10.1177/1747021818776145 29706121

[24] Matsuyoshi, D. & Watanabe, K. People have modest, not good, insight into their face recognition ability: A comparison between self-report questionnaires. *Psychol. Res.* **85**, 1713–1723 (2021). 32436049 10.1007/s00426-020-01355-8 PMC8211616

[25] Arizpe, J. M. *et al.* Self-reported face recognition is highly valid, but alone is not highly discriminative of prosopagnosia-level performance on objective assessments. *Behav. Res. Methods* **51**, 1102–1116 (2019). 30761463 10.3758/s13428-018-01195-w PMC6527346

[26] Burns, E. J., Gaunt, E., Kidane, B., Hunter, L. & Pulford, J. A new approach to diagnosing and researching developmental prosopagnosia: Excluded cases are impaired too. *Behav. Res. Methods.*10.3758/s13428-022-02017-w (2022). 36459376 10.3758/s13428-022-02017-w PMC9718472

[27] Shah, P., Sowden, S., Gaule, A., Catmur, C. & Bird, G. The 20 item prosopagnosia index (PI20): Relationship with the Glasgow face-matching test. *Royal Society Open Sci.* **2**, e150305 (2015). 10.1098/rsos.150305 PMC4680610 26715995

[28] Minio-Paluello, I., Porciello, G., Pascual-Leone, A. & Baron-Cohen, S. Face individual identity recognition: A potential endophenotype in autism. *Mol. Autism* **11**, 1–16 (2020). 33081830 10.1186/s13229-020-00371-0 PMC7576748

[29] Carpenter, K. L. & Williams, D. M. A meta-analysis and critical review of metacognitive accuracy in autism. *Autism* **27**, 512–525 (2023). 35796111 10.1177/13623613221106004

[30] Schönbrodt, F. D. & Perugini, M. At what sample size do correlations stabilize?. *J. Res. Personality* **47**, 609–612 (2013). 10.1016/j.jrp.2013.05.009

[31] Murray, E. & Bate, S. Diagnosing developmental prosopagnosia: Repeat assessment using the Cambridge Face Memory Test. *Royal Society Open Sci.* **7**, e200884 (2020). 10.1098/rsos.200884 PMC7540801 33047048

[32] Keating, C. T., Fraser, D. S., Sowden, S. & Cook, J. L. Differences between autistic and non-autistic adults in the recognition of anger from facial motion remain after controlling for alexithymia. *J. Autism Develop. Disord.* **52**, 1855–1871 (2022). 10.1007/s10803-021-05083-9 PMC8159724 34047905

[33] Walker, D. L., Palermo, R., Callis, Z. & Gignac, G. E. The association between intelligence and face processing abilities: A conceptual and meta-analytic review. *Intelligence* **96**, e101718 (2023). 10.1016/j.intell.2022.101718

[34] Bird, G. & Cook, R. Mixed emotions: The contribution of alexithymia to the emotional symptoms of autism. *Transl. Psychiatry* **3**, e285 (2013). 23880881 10.1038/tp.2013.61 PMC3731793

[35] Gehdu, B. K., Tsantani, M., Press, C., Gray, K. L. & Cook, R. Recognition of facial expressions in autism: Effects of face masks and alexithymia. *Quart. J. Exp. Psychol*. e17470218231163007 (2023). 10.1177/17470218231163007 36872641

[36] Thoma, P., Soria Bauser, D., Edel, M. A., Juckel, G. & Suchan, B. Configural processing of emotional bodies and faces in patients with attention deficit hyperactivity disorder. *J. Clin. Exp. Neuropsychol.* **42**, 1028–1048 (2020). 33161842 10.1080/13803395.2020.1840521

[37] Seernani, D. *et al.* Social and non-social gaze cueing in autism spectrum disorder, attention-deficit/hyperactivity disorder and a comorbid group. *Biol. Psychol.* **162**, e108096 (2021). 10.1016/j.biopsycho.2021.108096 33891995

[38] Baron-Cohen, S., Wheelwright, S., Skinner, R., Martin, J. & Clubley, E. The autism-spectrum quotient (AQ): Evidence from asperger syndrome/high-functioning autism, malesand females, scientists and mathematicians. *J. Autism Develop. Disorders* **31**, 5–17 (2001). 10.1023/A:1005653411471 11439754

[39] Bagby, R. M., Parker, J. D. & Taylor, G. J. The twenty-item Toronto Alexithymia Scale-I. Item selection and cross-validation of the factor structure. *J. Psychosomatic Res.* **38**, 23–32 (1994). 10.1016/0022-3999(94)90005-1 8126686

[40] Taylor, G. J., Bagby, R. M. & Parker, J. D. The 20-Item Toronto Alexithymia Scale: IV. Reliability and factorial validity in different languages and cultures. *J. Psychosomatic Res.* **55**, 277–283 (2003). 10.1016/S0022-3999(02)00601-3 12932803

[41] Kessler, R. C. *et al.* The World Health Organization Adult ADHD Self-Report Scale (ASRS): A short screening scale for use in the general population. *Psychol. Med.* **35**, 245–256 (2005). 15841682 10.1017/S0033291704002892

[42] Kessler, R. C. *et al.* Validity of the World Health Organization Adult ADHD Self-Report Scale (ASRS) Screener in a representative sample of health plan members. *Int. J. Methods Psychiatric Res.* **16**, 52–65 (2007). 10.1002/mpr.208 PMC2044504 17623385

[43] Chierchia, G. *et al.* The matrix reasoning item bank (MaRs-IB): Novel, open-access abstract reasoning items for adolescents and adults. *Royal Society Open Sci.* **6**, 190232 (2019). 10.1098/rsos.190232 PMC6837216 31824684

[44] Anwyl-Irvine, A. L., Massonnié, J., Flitton, A., Kirkham, N. & Evershed, J. K. Gorilla in our midst: An online behavioral experiment builder. *Behav. Res. Methods* **52**, 388–407 (2020). 31016684 10.3758/s13428-019-01237-x PMC7005094

[45] JASP-Team. JASP (Version 0.16.3)[Computer software]. *Amsterdam, The Netherlands.* (2022).

[46] Jeffreys, H. *Theory of probability (3rd ed.)*. (Oxford University Press, 1961).

[47] Hours, C., Recasens, C. & Baleyte, J. M. ASD and ADHD comorbidity: What are we talking about?. *Front. Psychiatry* **13**, e154 (2022). 10.3389/fpsyt.2022.837424 PMC8918663 35295773

[48] Leitner, Y. The co-occurrence of autism and attention deficit hyperactivity disorder in children–what do we know? *Front. Human Neurosci*. **8** (2014). 10.3389/fnhum.2014.00268 PMC4010758 24808851

[49] Tsantani, M., Gray, K. L. H. & Cook, R. New evidence of impaired expression recognition in developmental prosopagnosia. *Cortex* (2022). 10.1016/j.cortex.2022.05.008 35728295

[50] Cuve, H. C. *et al.* Alexithymia explains atypical spatiotemporal dynamics of eye gaze in autism. *Cognition* **212** (2021). 10.1016/j.cognition.2021.104710 33862441

[51] Gray, K. L. H. & Cook, R. Should developmental prosopagnosia, developmental body agnosia, and developmental object agnosia be considered independent neurodevelopmental conditions?. *Cognit. Neuropsychol.* **35**, 59–62 (2018). 29658410 10.1080/02643294.2018.1433153

[52] Kracke, I. Developmental prosopagnosia in Asperger syndrome: Presentation and discussion of an individual case. *Develop. Med. Child Neurol.* **36**, 873–886 (1994). 7926319 10.1111/j.1469-8749.1994.tb11778.x

[53] Conti-Ramsden, G., Simkin, Z. & Botting, N. The prevalence of autistic spectrum disorders in adolescents with a history of specific language impairment (SLI). *J. Child Psychol. Psychiatry* **47**, 621–628 (2006). 16712639 10.1111/j.1469-7610.2005.01584.x

[54] Dziuk, M. A. *et al.* Dyspraxia in autism: Association with motor, social, and communicative deficits. *Develop. Med. Child Neurol.* **49**, 734–739 (2007). 17880641 10.1111/j.1469-8749.2007.00734.x

[55] Gilger, J. W. & Kaplan, B. J. Atypical brain development: A conceptual framework for understanding developmental learning disabilities. *Develop. Neuropsychol.* **20**, 465–481 (2001). 10.1207/S15326942DN2002_2 11892948

[56] DeGutis, J. *et al.* What is the prevalence of developmental prosopagnosia? An empirical assessment of different diagnostic cutoffs. *Cortex* **161**, 51–64 (2023). 36905701 10.1016/j.cortex.2022.12.014 PMC10065901

[57] Cook, R., Brewer, R., Shah, P. & Bird, G. Alexithymia, not autism, predicts poor recognition of emotional facial expressions. *Psychol. Sci.* **24**, 723–732 (2013). 23528789 10.1177/0956797612463582

[58] Bird, G., Press, C. & Richardson, D. C. The role of alexithymia in reduced eye-fixation in autism spectrum conditions. *J. Autism Develop. Disorders* **41**, 1556–1564 (2011). 10.1007/s10803-011-1183-3 21298331

[59] Ferri, S. L., Abel, T. & Brodkin, E. S. Sex differences in autism spectrum disorder: A review. *Curr. Psychiatry Rep.* **20**, 1–17 (2018). 29504047 10.1007/s11920-018-0874-2 PMC6477922

[60] Rødgaard, E. M., Jensen, K., Miskowiak, K. W. & Mottron, L. Representativeness of autistic samples in studies recruiting through social media. *Autism Res.* **15**, 1447–1456 (2022). 35809003 10.1002/aur.2777 PMC9541916

[61] Germine, L., Duchaine, B. & Nakayama, K. Where cognitive development and aging meet: Face learning ability peaks after age 30. *Cognition* **118**, 201–210 (2011). 21130422 10.1016/j.cognition.2010.11.002

[62] Gray, K. L. H., Biotti, F. & Cook, R. Evaluating object recognition ability in developmental prosopagnosia using the Cambridge Car Memory Test. *Cognitive Neuropsychol.* **36**, 89–96 (2019). 10.1080/02643294.2019.1604503 30973292

[63] Maniscalco, B. & Lau, H. A signal detection theoretic approach for estimating metacognitive sensitivity from confidence ratings. *Consciousness Cognition* **21**, 422–430 (2012). 22071269 10.1016/j.concog.2011.09.021

[64] Fleming, S. M. & Lau, H. C. How to measure metacognition. *Front. Human Neurosci.* **8**, e443 (2014). 10.3389/fnhum.2014.00443 PMC4097944 25076880

