# Supplementary material for: Autistic adults have insight into their relative face recognition ability
Source: Sci Rep. 2024 Aug 1;14:17802. doi: 10.1038/s41598-024-67649-8 (PMC11294533; doi:10.1038/s41598-024-67649-8)
Supplement: Supplementary file 1 — Supplementary Information. [file 41598_2024_67649_MOESM1_ESM.docx]

**Autistic adults have insight into their relative face recognition ability**

**Bayparvah Kaur Gehdu, Clare Press, Katie L. H. Gray, Richard Cook**

**Supplementary Material**

In the main text, we describe a Matrix Reasoning Task (MRT) that was used to estimate individual differences in participants’ non-verbal intelligence. The MRT consisted of forty items, with scores ranging from 0 to 40 (chance performance = 10). Participants were given 30 seconds to complete each puzzle by selecting the correct answer from 4 options. Participants responded using keyboard number keys (1-4), were given a 5-second warning before the end of each trial, and received no feedback. Each participant attempted all forty items. Participants had to complete 3 practice trials correctly before beginning the test.

To assess the test-retest reliability of this measure, 100 non-autistic participants (*M*_age_ = 34.90 years, *SD*_age_ = 10.16 years; 27 males, 73 females) were recruited through [www.prolific.co](http://www.prolific.co) and asked to complete the test twice. The average interval between the first and second attempt was 170.2 days (range: 75 days to 297 days), during which time participants were given no feedback regarding their level of performance. The scores seen at the first attempt (*M* = 26.07, *SD* = 5.714) and second attempt (*M* = 26.13, *SD* = 6.530) did not differ significantly [*t*(99) = .131, *p* = .896]. We observed strong positive correlation (Pearson’s *r*) between the two sets of scores [*N* = 100, *r*_p_ = .727, *p* < .001] (Figure S1).


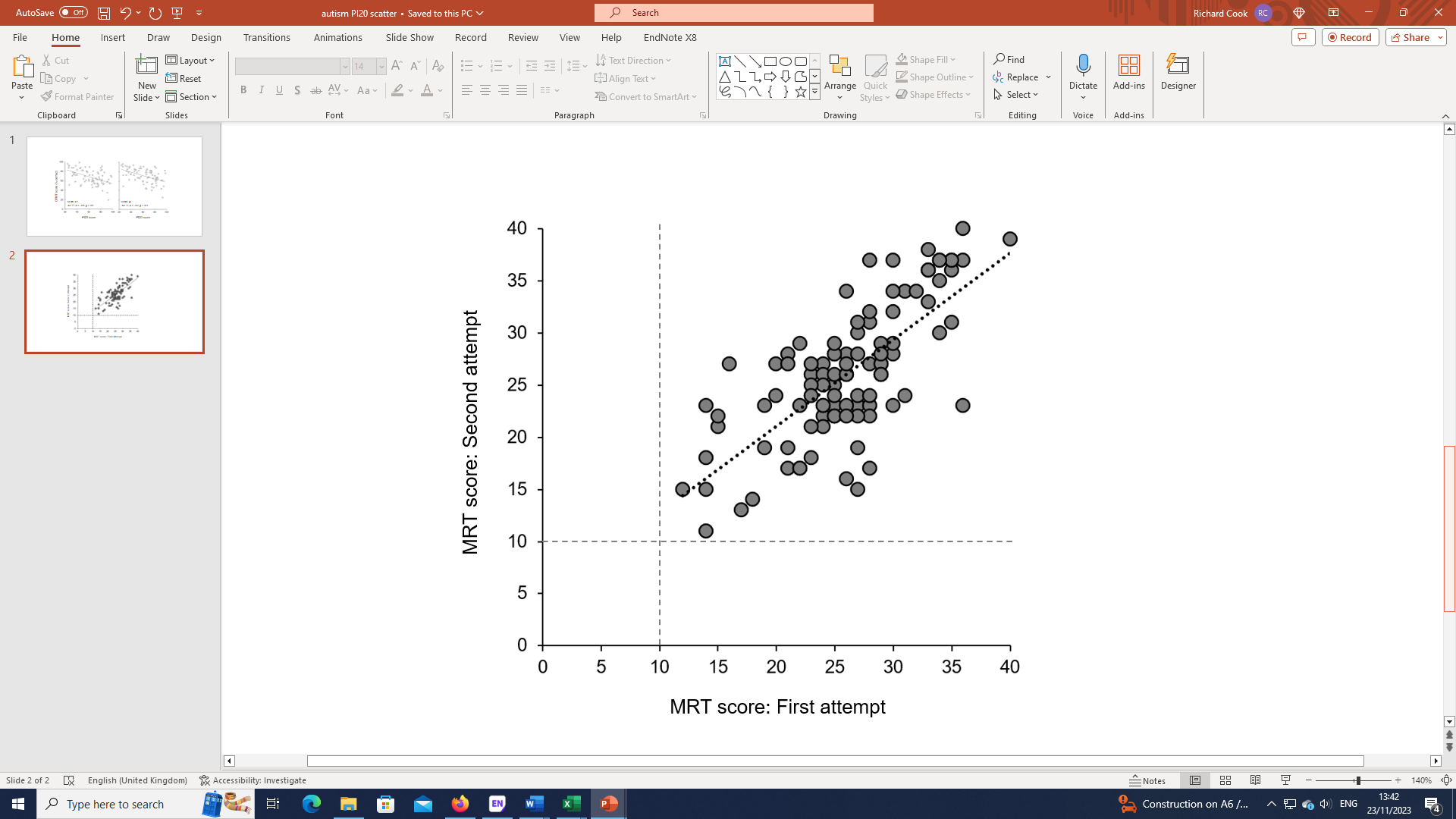


**Figure S1**. Scatterplot illustrating participants’ scores on the Matrix Reasoning Task (MRT) at the first and second attempt.

**Table S1.** The statements comprising the PI20.

| 1. My face recognition ability is worse than most people |
| --- |
| 2. I have always had a bad memory for faces |
| 3. I find it notably easier to recognize people who have distinctive facial features |
| 4. I often mistake people I have met before for strangers |
| 5. When I was at school, I struggled to recognize my classmates |
| 6. When people change their hairstyle or wear hats, I have problems recognizing them |
| 7. I sometimes have to warn new people I meet that I am ‘bad with faces’ |
| 8. I find it easy to picture individual faces in my mind* |
| 9. I am better than most people at putting a ‘name to a face’* |
| 10. Without hearing people's voices, I struggle to recognize them |
| 11. Anxiety about face recognition has led me to avoid certain social or professional situations |
| 12. I have to try harder than other people to memorize faces |
| 13. I am very confident in my ability to recognize myself in photographs* |
| 14. I sometimes find movies hard to follow because of difficulties recognizing characters |
| 15. My friends and family think I have bad face recognition or bad face memory |
| 16. I feel like I frequently offend people by not recognizing who they are |
| 17. It is easy for me to recognize individuals in situations that require people to wear similar clothes (e.g. suits, uniforms and swimwear)* |
| 18. At family gatherings, I sometimes confuse individual family members |
| 19. I find it easy to recognize celebrities in ‘before-they-were-famous’ photos, even if they have changed considerably* |
| 20. It is hard to recognize familiar people when I meet them out of context (e.g. meeting a work colleague unexpectedly while shopping) |

N.B. Asterisks indicate items that are reverse scored.
